# Novel Weed-Extracted Silver Nanoparticles and Their Antibacterial Appraisal against a Rare Bacterium from River and Sewage Treatment Plan

**DOI:** 10.3390/nano8010009

**Published:** 2017-12-26

**Authors:** Achmad Syafiuddin, Salmiati, Tony Hadibarata, Ahmad Beng Hong Kueh, Mohd Razman Salim

**Affiliations:** 1 Department of Environmental Engineering, Faculty of Civil Engineering, Universiti Teknologi Malaysia, Johor 81310, Malaysia; udenfisika@gmail.com (A.S.); mohdrazman@utm.my (M.R.S.); 2 Centre for Environmental Sustainability and Water Security (IPASA), Research Institute for Sustainable Environment, Faculty of Civil Engineering, Universiti Teknologi Malaysia, Johor 81310, Malaysia; 3 Department of Environmental Engineering, Faculty of Engineering and Science, Curtin University, Miri 98009, Sarawak, Malaysia; hadibarata@curtin.edu.my; 4 Construction Research Centre (CRC), Institute for Smart Infrastructure and Innovative Construction (ISIIC), Faculty of Civil Engineering, Universiti Teknologi Malaysia, Johor 81310, Malaysia; kbhahmad@utm.my

**Keywords:** silver nanoparticles, green synthesis, *Chromobacterium haemolyticum*, weed extracts

## Abstract

This is the first investigation to demonstrate the use of biochemical contents present within *Cyperus rotundus*, *Eleusin indica*, *Euphorbia hirta*, *Melastoma malabathricum*, *Clidemia hirta* and *Pachyrhizus erosus* extracts for the reduction of silver ion to silver nanoparticles (AgNPs) form. In addition, the antibacterial capability of the synthesized AgNPs and plant extracts alone against a rare bacterium, *Chromobacterium haemolyticum* (*C. haemolyticum*), was examined. Moreover, ultraviolet-visible spectroscopy (UV-vis), Fourier transform infrared spectroscopy (FTIR), field emission scanning electron microscopy (FESEM), energy-dispersive X-ray spectroscopy (EDX) and inductively coupled plasma atomic emission spectroscopy (ICPOES) of the synthesized AgNPs were characterized. The smallest AgNPs can be produced when *Cyperus rotundus* extracts were utilized. In addition, this study has found that the synthesis efficiencies using all plant extracts are in the range of 72% to 91% with the highest percentage achieved when *Eleusin indica* extract was employed. All synthesized AgNPs have antibacterial capability against all examined bacteria depending on their size and bacteria types. Interestingly, *Melastoma malabathricum* and *Clidemia hirta* extracts have demonstrated an antibacterial ability against *C. haemolyticum*.

## 1. Introduction

The rapid expanding of works carrying the theme of nanomaterials in the past several years is widely evidenced in the literature [1]. Since it is well-recognized that nanomaterials possess extraordinary features in terms of physical, chemical and biological compared to their larger form, they have been hugely explored for characterization and application. Up to the present time, nanoparticle production remains as one of the central issues in the nanomaterial research. To name a few, widely examined nanoparticle types include iron, copper, silicon, gold and silver [2,3,4,5,6]. Among these, silver nanoparticles (AgNPs) have been extensively investigated because of their appealing properties [7]. Therefore, AgNPs have been utilized for various applications such as electronics, sensors and recently widely explored for medical devices. 

The exploration of AgNPs for medical fields particularly as an antibacterial agent has been extensively carried out. To explore their application, the antibacterial capability of AgNPs against several bacteria has been ratified [8,9,10,11,12,13]. The role of the antibacterial capability of AgNPs in terms of the surface science has been well reviewed by Le Ouay and Stellacci [14]. The specific effect of the nanoparticulate objects on these nanoparticles such as adsorption at bacterial surfaces can significantly improve their antibacterial capability. In addition, the role of Ag^+^ release has been found possible. In general, the antibacterial capability of AgNPs is crucially affected by their surface physical and chemical properties as well as bacteria types. Therefore, investigation for effects on new potential bacteria is urgently needed.

Since the physical and chemical approaches were ratified to have numerous constraints in terms of time consumption, cost and toxicity, recent synthesis developments have focused on how these nanoparticles can be greenly synthesized using natural resources such as plant extracts. Synthesis methods for AgNPs production by means of roots, seeds, fruits and leaves are well-studied. Accessibility of roots and seeds is relatively more difficult compared to leaves and fruits. Fruits and leaves are still recognized as the vitamin and vegetable sources for developing countries including Indonesia and Malaysia. To overcome these matters, an alternative natural resource that is abundantly available is urgently needed. In this respect, the application of grass weeds seems strategic for the explorative purpose. In addition, a huge usage of weeds may also support the enhancement of the environmental quality since they are well-known as plants with a negative impact on agriculture particularly by monopolizing resources. 

*Cyperus rotundus*, sometimes called the coco-grass, Java grass or nut grass, is a species of Cyperaceae. It is also labeled as the worst weed that can damage ecosystem services and monopolize resources [15]. Similarly, negatively impactful is *Eleusin indica*, which can be categorized as the species of grass in the Poaceae family and is commonly used for animals’ food. *Euphorbia hirta* can be categorized as a pantropical weed that is distributed in the tropical regions such as India, Malaysia and Indonesia. It is also well-established as traditional medicine with analgesic and anti-inflammatory properties [16]. *Melastoma malabathricum* origin locations are in Asia, Polynesia and Australia. It is also known as a problematic weed that commonly forms the dense thickets. *Clidemia hirta* can be categorized as an invasive plant species in the tropical regions. Although it originated from the American Neotropics, *Clidemia hirta* is also widely distributed in many tropical countries such as Indonesia and Malaysia. In addition, it is also found to cause significant environmental damage, highly invasive and has the potential to alter forest regeneration [17]. *Pachyrhizus erosus* is believed to be originated from Mexico and Central America and is presently widespread in the tropical Asia. 

The intensive use of the plant extracts of weeds is due to their internal biomolecule such as protein, terpenoids and flavonoids, which offer potential as a bioreductant to reduce metal ions to form AgNPs [15,18,19,20,21,22,23,24,25]. For instance, the main terpenoids, such as eugenol, present within *Cinnamomum zeylanisum* extracts has been ratified as the bioreduction agent of gold(III) chloride trihydrate (HAuCl_4_) and silver nitrate (AgNO_3_) in forming nanoparticle [26]. The dissociation of proton of the eugenol OH-group is believed to result in the formation of resonance structures that are required for further oxidation to reduce the metal ion [26,27]. This process can be correlated to the active reduction of metal ions to form the nanoparticle. In the flavonoids philosophy, reducing metal ions to form nanoparticles was facilitated by the flavonoids transformation from the enol-form to the keto-form that can release a reactive hydrogen atom [28]. Therefore, the present work aims to evaluate the application of biochemical contents present within *Cyperus rotundus*, *Eleusin indica*, *Euphorbia hirta*, *Melastoma malabathricum*, *Clidemia hirta* and *Pachyrhizus erosus* extracts to synthesize AgNPs from AgNO_3_. In addition, an antibacterial capability of the synthesized AgNPs and plant extracts alone against *C. haemolyticum* isolated from the sewage treatment plant (STP) and the river was also evaluated in this study. *Chromobacterium haemolyticum* (*C. haemolyticum*) can be categorized as new Gram-negative bacteria, firstly proposed in 2008 by Han et al. [29]. To our best knowledge, the antibacterial capability of AgNPs against these bacteria has not been reported elsewhere. The outcomes are highly valuable for future applications in water purification and medicine. 

## 2. Results and Discussion

### 2.1. Ultraviolet-Visible Spectroscopy (UV-Vis)

UV-vis spectroscopy monitors the absorption in the ultraviolet-visible spectral region and is commonly used to characterize plasmonic properties of synthesized nanoparticles. Studies have demonstrated that AgNPs absorb the spectrum in the visible region of around 400 nm due to the excitation of the localized surface Plasmon resonance [30,31,32,33]. Since this property is highly affected by nanoparticle size and the surrounding media, it is also possible that AgNPs have the plasmon band of around 500 nm or slightly higher [34]. Several researchers have evaluated the correlation between the UV-vis spectrum and nanoparticle properties. For instance, uniform spherical AgNPs can be associated with a single peak in the UV-vis spectrum while AgNPs in the irregular shapes have two or more peaks depending on their symmetry [35]. In addition, increase in nanoparticles size can be identified by their maximum absorbance located at the higher wavelength range [36]. Moreover, increasing the absorbance spectra indicates a higher production of AgNPs [37]. 

**Figure 1:**
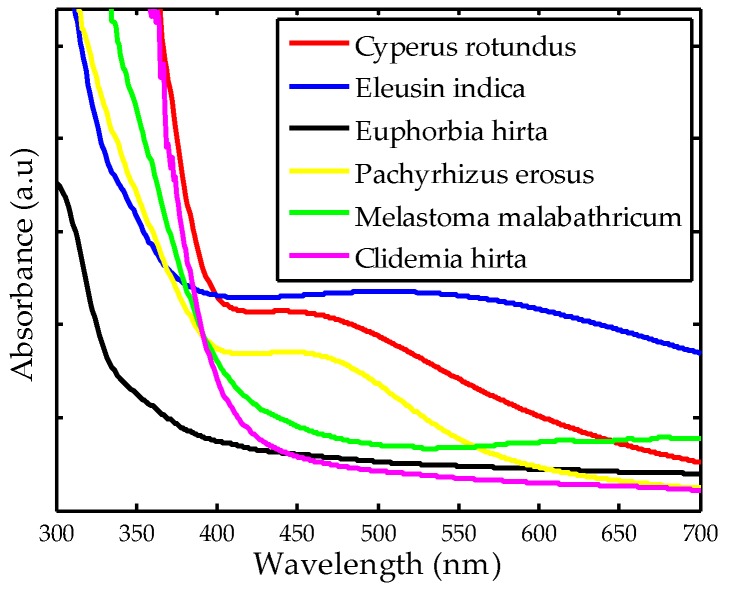

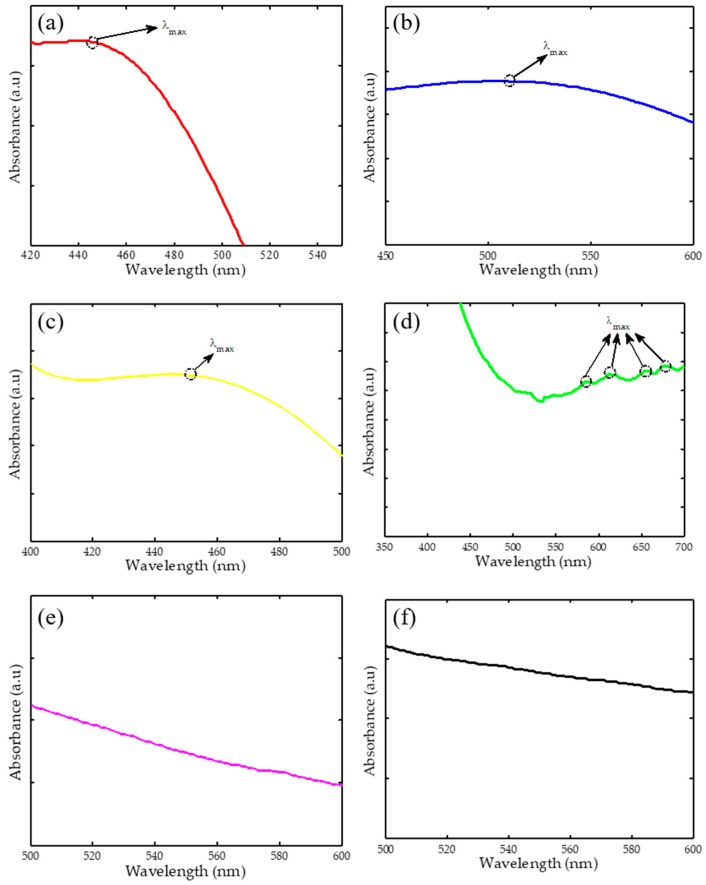
Ultraviolet-visible spectroscopy (UV-vis spectra) of AgNPs synthesized using different plant extracts. (**a**–**f**) are zoomed in portion of each spectrum.

Figure 1 shows the UV-vis spectra of AgNPs synthesized using currently plant extracts. It has been found that AgNPs synthesized using *Cyperus rotundus*, *Eleusin indica* and *Pachyrhizus erosus* have single maximum peaks at 446, 524 and 452, respectively, suggesting that they are spherical in shapes. On the other hand, there is an absence of a clear maximum peak for AgNPs synthesized using *Clidemia hirta* and *Euphorbia hirta*, indicating they are of irregular shape or larger nanoparticle sizes [35]. In addition, AgNPs synthesized using *Melastoma malabathricum* have more than one peak in the wavelength range of 550 to 780 nm, implying irregularity in their nanoparticle shapes [35]. In the case of the spherical nanoparticle, it is found that AgNPs synthesized using *Cyperus rotundus* had the maximum absorbance located at the lower wavelength range as shown in Figure 1. This finding suggests that smaller sizes can be produced when *Cyperus rotundus* was employed [36]. For the irregular nanoparticle shape, it is rather difficult to make a direct comparison since there are more than one maximum peak spectra. Moreover, AgNPs synthesized using *Eleusin indica* exhibited the highest UV-vis spectra absorbance compared to others, indicating more AgNPs were produced [37].

**Figure 2:**
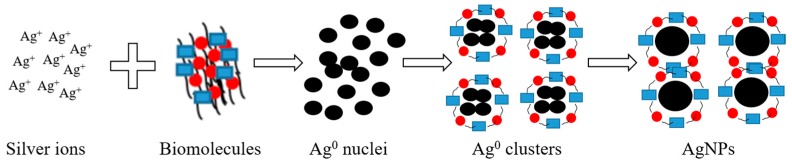
A schematic presentation of AgNPs synthesized using biomolecules extracted from plants.

The reduction of metal ion to the nanoparticle form using biochemical contents present within the presently employed plant extracts is indicated by the change in solution color from yellow to brown or reddish yellow to deep red. In the synthesis, AgNO_3_ was dissociated into Ag^+^ and NO_3_^−^. Phenolic compounds present within all the presently investigated plant extracts bind and therefore reduce the metal salt into a nanoparticle form. It was experimentally observed that the reduction of metal ions to form nanoparticles is facilitated by the flavonoids transformation from the enol-form to the keto-form, resulting in the release of a reactive hydrogen atom [28]. The biomolecules attached to the nanoparticles prevent their agglomeration. The advantage of using biomolecules contained within the plants extract is that they not only act as a reducing agent but also as a stabilizing agent, which is difficult to handle when the chemical procedure is employed. For a comprehensive take on the synthesis, a schematic presentation of the reduction of silver ion to the formation of the nanoparticle using biochemicals present within all presently used plant extracts is shown in Figure 2. 

### 2.2. Fourier Transform Infrared Spectroscopy (FTIR) 

**Figure 3:**
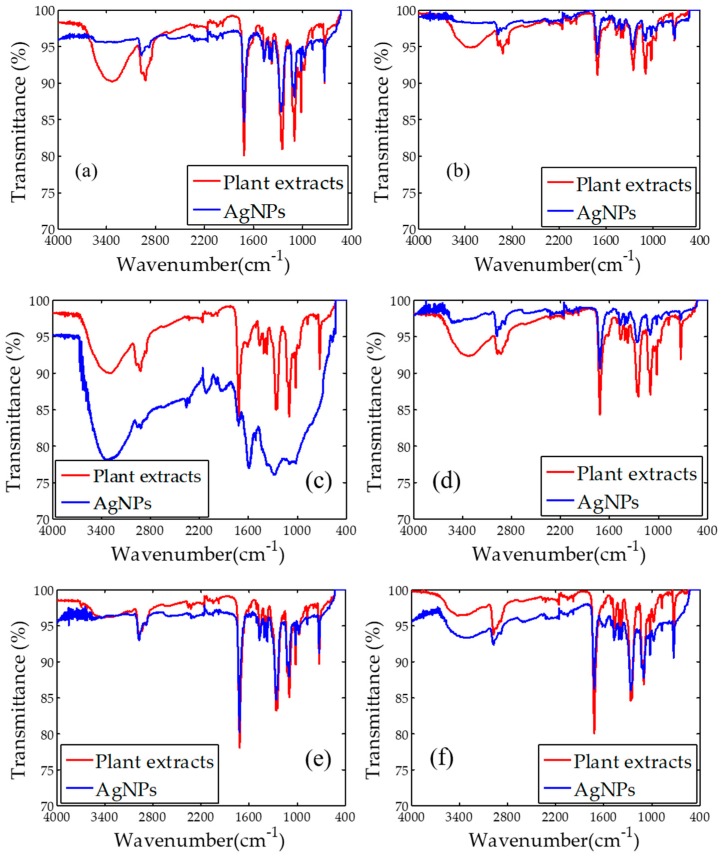
Fourier transform infrared spectroscopy (FTIR spectra) of plant extracts and AgNPs synthesized using (**a**) *Cyperus rotundus*; (**b**) *Eleusin indica*; (**c**) *Euphorbia hirta*; (**d**) *Melastoma malabathricum*; (**e**) *Clidemia hirta*; and (**f**) *Pachyrhizus erosus.*

FTIR spectra of AgNPs synthesized using all plant extracts are presented in Figure 3. In general, the synthesized AgNPs have 5 to 6 prominent peaks in the FTIR spectra. There are little changes in terms of intensity and wavenumber at the maximum peak of the FTIR spectra between the plant extracts and synthesized AgNPs. Specifically, AgNPs synthesized using *Cyperus rotundus* have peaks at 2976, 1715, 1248, 1099 and 726 cm^−1^ characterized as –CH, C=O, C–O–C, P–O and N–H stretching vibrations, respectively [38]. The peak at 3363 correlated to –OH stretching vibration contained in *Cyperus rotundus* extracts disappeared in the FTIR spectra of AgNPs synthesized by their extracts. In addition, C–O–C stretching vibration for AgNPs slightly changed from 1248 (*Cyperus rotundus* extracts) to 1262 cm^−1^. For AgNPs synthesized using *Eleusin indica*, peaks at 3339 and 2919 cm^−1^, categorized as OH and –CH did not arise. Also, N–H stretching changed from 726 (*Eleusin indica* extracts) to 721 cm^−1^ (AgNPs). Alternatively, N–H stretching appeared from *Euphorbia hirta* also disappeared in AgNPs synthesized by their extracts. When *Melastoma malabathricum* was employed as reducing and stabilizing agents, –OH stretching contained in their extracts cannot be observed in the synthesized AgNPs. Moreover, several prominent peaks of *Clidemia hirta* and *Pachyrhizus erosus* were exhibited at the FTIR spectra for AgNPs synthesized using their extracts as shown in Figure 3. For a complete overview, the biochemical contents present within the presently employed plants are summarized in Table 1. Also, possible compounds of AgNPs synthesized using different plant extracts are given in Table 2. In general, it is interesting to see from this work that the biomolecules contained within the plant extracts exist in the synthesized AgNPs. 

### 2.3. Field Emission Scanning Electron Microscopy (FESEM)

**Figure 4:**
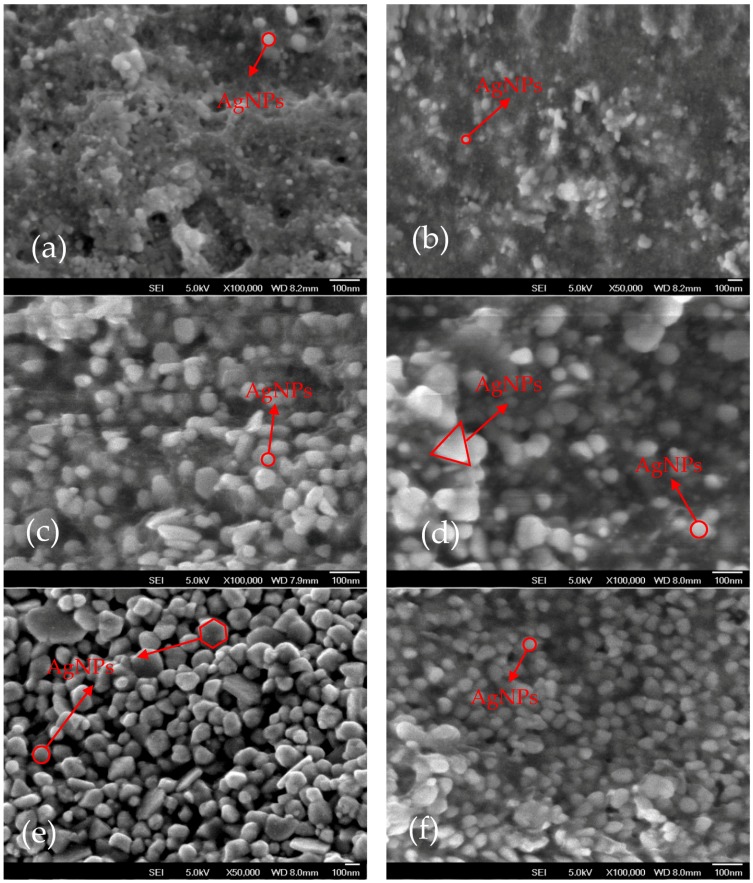
FESEM of AgNPs synthesized using (**a**) *Cyperus rotundus*; (**b**) *Eleusin indica*; (**c**) *Euphorbia hirta*; (**d**) *Melastoma Malabathricum*; (**e**) *Clidemia hirta*; and (**f**) *Pachyrhizus erosus*.

FESEM of AgNPs synthesized using all plant extracts is shown in Figure 4. In addition, Table 3 provides the summary of shape and size of AgNPs synthesized using all plant extracts. It is evidenced that AgNPs synthesized using *Cyperus rotundus*, *Eleusin indica* and *Pachyrhizus erosus* are spherical in shape with the sizes of 20.5 ± 9.6, 55.0 ± 24.1 and 40.6 ± 10.8 nm, respectively. These findings support the UV-vis characteristics exhibiting that AgNPs synthesized using their extracts have a single peak. When *Euphorbia hirta* and *Clidemia hirta* were used to synthesize AgNPs, AgNPs with irregular shapes with the sizes of 56.25 ± 21.8 and 57.4 ± 24.2 nm, respectively, were produced. Alternatively, irregular shapes AgNPs around 108.35 ± 36.3 nm were also obtained by applying *Melastoma malabathricum* extracts as a bioreduction agent. The result also matches with the UV-vis spectra showing more than single peak or no obvious peak when AgNPs were synthesized using *Euphorbia hirta*, *Clidemia hirta* and *Melastoma malabathricum*. This study has also confirmed that the smallest nanoparticles can be produced when *Cyperus rotundus* is employed as the reducing and stabilizing agents (See Figure 4 and Table 3). 

### 2.4. Energy-Dispersive X-ray Spectroscopy EDX

**Figure 5:**
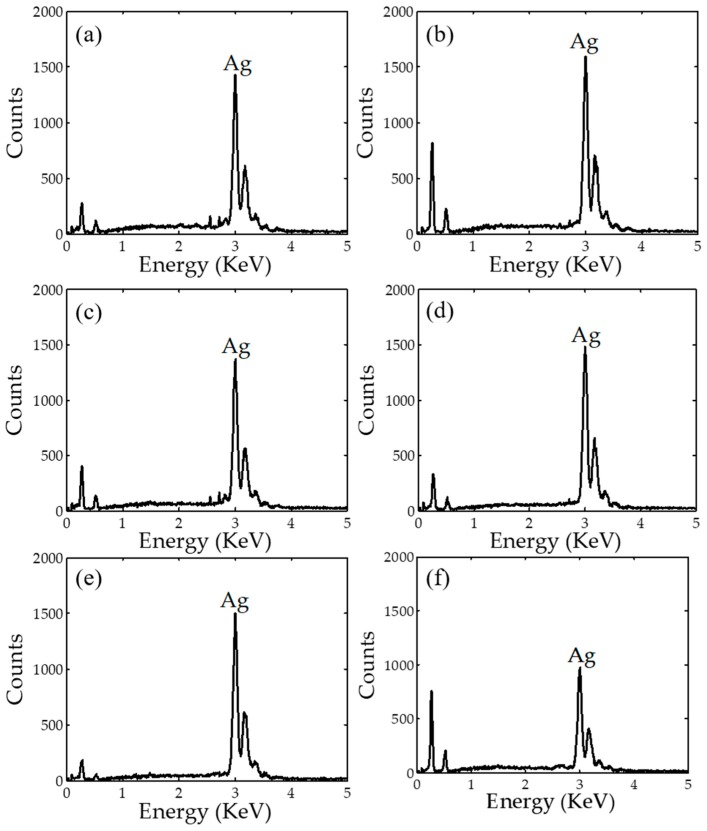
Energy-dispersive X-ray spectroscopy (EDX spectra) of AgNPs synthesized using (**a**) *Cyperus rotundus*; (**b**) *Eleusin indica*; (**c**) *Euphorbia hirta*; (**d**) *Melastoma Malabathricum*; (**e**) *Clidemia hirta*; and (**f**) *Pachyrhizus erosus*.

EDX spectra of AgNPs synthesized by all plant extracts are displayed in Figure 5. It is clear that all synthesized AgNPs exhibit the EDX peak at approximately 3 keV because of their surface Plasmon resonance [39]. The highest peaks in Figure 5 confirm that the metal AgNPs are the dominant element in the samples. This observation is validated by the percentages of silver, which are in the range of 69–94% compared to other elements. In addition, other elements such as carbon and oxygen possibly exist due to the presence of bio-organic molecules produced in the synthesis process, which is also reported elsewhere [7,38]. 

### 2.5. Inductively Coupled Plasma Atomic Emission Spectroscopy (ICPOES)

**Figure 6:**
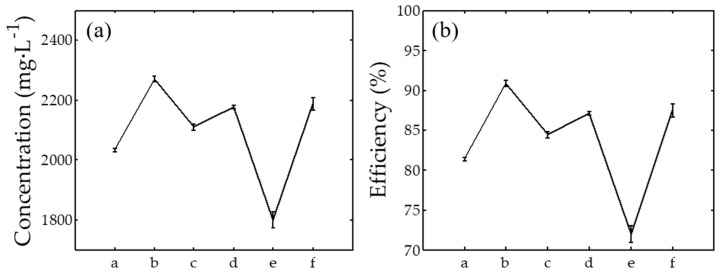
(**a**) Concentration of AgNPs and (**b**) their synthesis efficiency. a–f at the x-axis refer to AgNPs synthesized using *Cyperus rotundus*, *Eleusin indica*, *Euphorbia hirta*, *Melastoma Malabathricum*, *Clidemia hirta*, and *Pachyrhizus erosus*, respectively.

One major challenge in the nanoparticle study is the method that can be employed to synthesize nanoparticles efficiently. To address this issue, several approaches have been proposed and continually revised. A combination of physical and chemical approaches was reported to successfully fabricate AgNPs efficiently up to 100 mmol·L^−1^ [40]. Another study has shown that the proposed procedure successfully produced about 20 mg AgNPs from 30 mg AgNO_3_ dissolved in ethylene glycol and poly(*N*-vinyl pyrrolidone) [41]. In this work, the synthesis efficiency was calculated by the following formula:

(1) $$\eta =\frac{C_{0}}{C_{i}}\times 100\%$$

where *C*_0_ is the AgNPs concentration and *C_i_* is the concentration of AgNO_3_ dissolved in the solution. Figure 6 shows the concentration of the synthesized AgNPs obtained from the ICPOES analysis. The present procedure has successfully produced AgNPs with high concentration ranging from 1800 to 2270 mg·L^−1^ from 2500 mg AgNO_3_ dissolved in the ultrapure water and plant extracts. In general, 72% to 91% of AgNO_3_ was successfully employed to produce AgNPs with the highest efficiency achieved when AgNPs were synthesized using *Eleusin indica* as shown also in Figure 6.

### 2.6. Antibacterial Appraisal

The antibacterial capability of AgNPs was evaluated using bacteria isolated from STP and rivers. Based on the phylogenetic position, the bacteria isolated from rivers and STP are close to *C*. *haemolyticum* and *Chromobacterium violaceum*. *Chromobacterium violaceum* can be categorized as the bacteria having violacein pigment or purple-colored dye [42]. However, the authors did not observe the pigment from the present bacteria. Therefore, this study categorized the present bacteria as *C*. *haemolyticum*. Moreover, *C*. *haemolyticum* UDIN1, *C*. *haemolyticum* UDIN2 and *C*. *haemolyticum* UDIN3 are the name of bacteria isolated from STP, river 1 and river 2, respectively. In addition, it was also tested against Gram-negative and -positive bacteria, namely, *E*. *coli* and *B*. *cereus*, respectively. 

**Figure 7:**
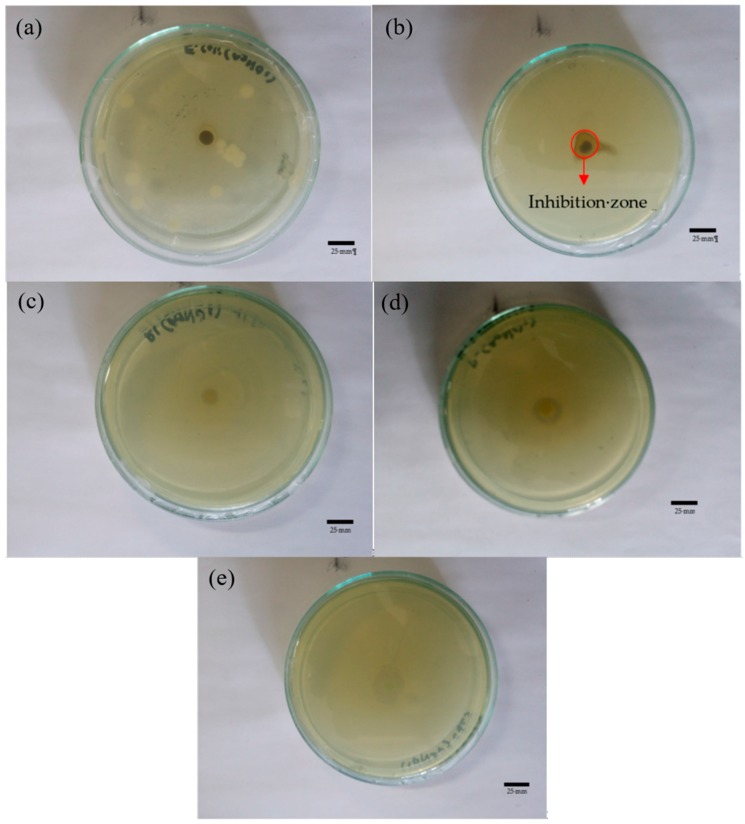
Zone of inhibition of *Cyperus rotundus* against (**a**) *E. coli*; (**b**) *B. cereus*; (**c**) *C. haemolyticum* UDIN2; (**d**) *C. haemolyticum* UDIN3; and (**e**) *C. haemolyticum* UDIN1. The scale bar is 25 mm.

**Figure 8:**
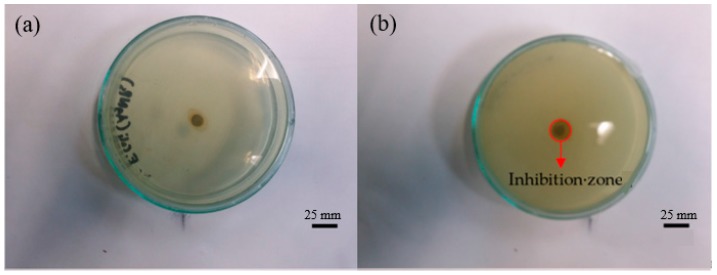

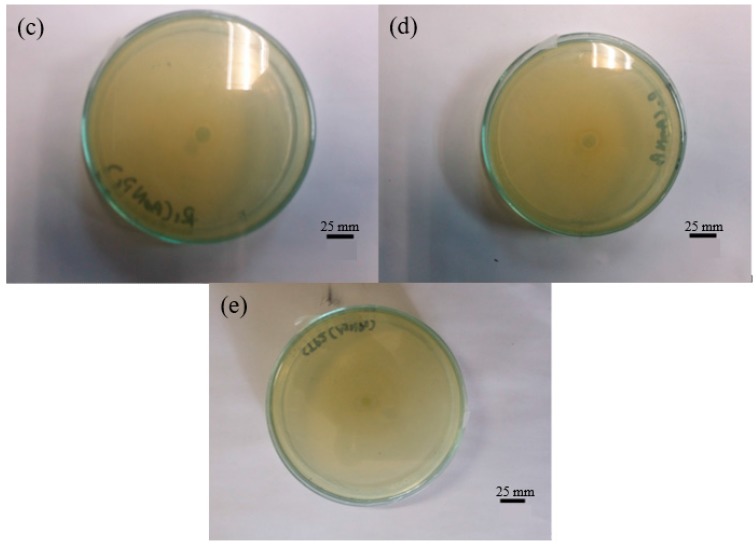
Zone of inhibition of AgNPs synthesized using *Euphorbia hirta* against (**a**) *E. coli*; (**b**) *B. cereus*; (**c**) *C. haemolyticum* UDIN2; (**d**) *C. haemolyticum* UDIN3; and (**e**) *C. haemolyticum* UDIN1.

Table 4 summarizes the zones of inhibition of the synthesized AgNPs, AgNO_3_, water and plant extracts against the presently considered bacteria. Figure 7 and Figure 8 show some examples of the zone of inhibition of the presently produced nanoparticles against all investigated bacteria. It was found that AgNPs synthesized using *Cyperus rotundus* produced the zones of inhibition ranging from 8.00 ± 0.26 to 15.00 ± 0.36 mm against all the studied bacteria. When AgNPs were synthesized using *Eleusin indica*, the zones of inhibition were in the range of 8.70 ± 0.06 to 12.30 ± 0.06 mm. In addition, it was found that AgNPs synthesized using *Melastoma malabathricum* inhibit the growth of all bacteria ranging from 1.17 ± 0.50 to 1.60 ± 0.10 mm. When AgNPs were synthesized using *Clidemia hirta*, the zones of inhibition were in the range of 1.03 ± 0.06 to 1.60 ± 0.10 mm. Moreover, AgNPs produced by applying *Pachyrhizus erosus* have zones of inhibition fluctuating from 9.00 ± 0.10 to 15.00 ± 0.10 mm. AgNO_3_ has been found to inhibit the growth of all studied bacteria ranging from 0.93 ± 0.12 to 1.19 ± 0.25 mm and determined to be the best antibacterial agent when used against *C. haemolyticum* UDIN3. By comparison, water provides zero inhibition against all bacteria.

It was ratified from this work that AgNPs synthesized using *Cyperus rotundus* had the highest zone of inhibition compared to others when they were applied against *E. coli*. AgNPs synthesized using *Pachyrhizus erosus* had the highest zone of inhibition compared to others when they were applied against *B. cereus*. In addition, AgNPs synthesized using *Pachyrhizus erosus* performed as the best antibacterial agent against all *C. haemolyticum*. Findings from this study revealed that antibacterial capability of AgNPs is different depending on their size and synthesis procedure. For instance, when *Cyperus rotundus* was employed to synthesize AgNPs, the best performance of the antibacterial capability of the produced nanoparticles is possibly due to the effect of nanoparticles size. It was previously discussed that the smallest nanoparticle size can be obtained when AgNPs were synthesized using this plant. It was reported from the previous work that the reduction of nanoparticle size from 100 to 5 nm was found to enhance the antibacterial capability of AgNPs [43]. This can be correlated to the enhancement in surface area to volume ratio with a decrease in particle size [43]. Although the size of AgNPs synthesized using *Pachyrhizus erosus* were slightly higher than that of *Cyperus rotundus*, their nanoparticle size was still smaller than those of *Eleusin indica*, *Euphorbia hirta*, *Melastoma Malabathricum* and *Clidemia hirta*. It is also possible that antibacterial capability of AgNPs was only affected by their nanoparticle size since various conditions such as surrounding media, bacteria types and other physio-chemical are crucial factors. 

The capability of AgNPs to inhibit bacteria is due to the interaction between AgNPs and the bacteria membrane once they are in contact with the organism [44]. Their interaction causes the bacteria membrane damage, which then leads to bacteria cellular death [44]. It is also possible via the oxidation of the silver nanoparticles to silver ions producing reactive oxygen species (ROS) [45]. In this particular mechanism, the toxicity properties are due to the involvement of disruption of the mitochondrial respiratory chain by AgNPs leading to the production of ROS, which in turn causes bacteria DNA damage [45]. Alternatively, it is possibly due to their nanoparticles surface charge. For instance, AgNPs having a negative charge on their surface are less toxic compared with those with a positive charge [46]. 

For a comprehensive overview, Table 5 lists the effects of AgNPs against various bacteria obtained from the previous works. From the table, it can be seen that AgNPs are able to inhibit various bacteria with different performance depending on their size and bacteria types. Nonetheless, it is noticeable that there is no publication reporting on the antibacterial activity of AgNPs against *C. haemolyticum*. Therefore, findings from the present work offer new contribution not only on the development of the reducing and stabilizing agents to synthesize nanoparticles but also the antibacterial capability against this bacteria type. The exploration of the present nanoparticles offers wide possibility as a new antibacterial solution for future medical treatment. 

## 3. Materials and Methods

### 3.1. Materials

*Cyperus rotundus*, *Eleusin indica*, *Euphorbia hirta*, *Melastoma malabathricum*, *Clidemia hirta* and *Pachyrhizus erosus* were obtained from the surrounding area of Universiti Teknologi Malaysia, Johor Bahru, Malaysia. AgNO_3_ was purchased from QReC, Auckland, New Zealand. Moreover, Wizard^®^ Genomic DNA Purification Kit (PMG-A1120) used in this study was obtained from Promega, Madison, WI, USA. 

### 3.2. Procedure of Plant Extraction

Firstly, 18 g fresh plant of weed type was weighed. For the extraction procedure, the plants were washed using the tap water followed by the ultrapure water three times each to completely remove any impurity. Each plant was then mixed with 200 mL ultrapure water in a 500 mL Erlenmeyer flask and boiled for 30 min. A pure plant extract was obtained from the mixture by means of filtering using a nylon membrane filter of 0.45 μm. The pure plant extracts were then stored in a fridge at a temperature of 7 °C pending for the next characterization. 

### 3.3. Synthesis of AgNPs

100 mL AgNO_3_ solution from the mixture of AgNO_3_ and ultrapure water with a concentration of 0.15 M in a 500 mL Erlenmeyer flask was prepared. Then, 100 mL plant extract was added slowly to the solution. The mixture was mechanically stirred at the rate of 100 rpm for 24 h in the dark condition. The mixture was then centrifuged at 8000× *g* for 45 min. The supernatant was then removed while the pellet was collected. The purification of AgNPs was done by cleansing the collected pellet using the ultrapure water at 8000× *g* for 45 min for three times. The purified pellet was subsequently air dried for 24 h to remove the water content. The synthesis procedure was performed in three replicates to ensure consistency in the result. The purified AgNPs produced using the present procedure were then stored at a temperature of 7 °C pending for the next investigation.

### 3.4. Water Sampling Location

Water samples were collected from Melana River (1.535869 N, 103.623216 E) and Sekudai River (1.542408 N, 103.662191 E), Johor Bahru, Malaysia. For brevity, Melana River and Sekudai River are abbreviated as river 1 and 2, respectively. In addition, a sample was also collected from the local STP (1.538247 N, 103.620674 E) at Desa Skudai, Johor Bahru, Malaysia. 

### 3.5. Bacteria Identification

A 500 μL water sample that contained unknown bacteria was pipetted into a 1.5 mL microcentrifuge tube and was centrifuged at 13,000× *g* for 2 min, where supernatant was then removed. Next, the lytic enzyme was added to the resuspended cell pellet in a total volume of 120 μL and followed by incubation at a temperature of 37 °C for 45 min. It was then centrifuged at 13,000× *g* for 2 min and the supernatant was removed. Subsequently, a 600 μL nuclei lysis solution was added to the mixture and incubated at a temperature of 80 °C for 5 min to lyse the cells. Addition of 3 μL of RNase solution to the cell lysate was conducted before incubating the mixture at 37 °C for 45 min. 200 μL of protein precipitation solution was next added to the RNase-treated cell lysate, with the whole mix incubated in ice for 5 min and centrifuged at 13,000× *g* for 3 min. The supernatant containing the DNA was transferred into a 1.5 mL microcentrifuge tube containing 600 μL of isopropanol and centrifuged at 13,000× *g* for 2 min. The supernatant was carefully poured and the tube drained on a clean absorbent paper. 600 μL of 70% ethanol was added and the tube was then gently inverted to wash the DNA pellet where all mixture centrifuged at 13,000× *g* for 2 min. A clean absorbent paper was again utilized to perform the draining of solution from the tube before allowing the pellet to air-dry for 10 min. 100 μL DNA rehydration solution was then added to the tube where the DNA was rehydrated by incubation at 65 °C for 1 h. The DNA was next stored at a temperature of 4 °C for further analysis. Subsequently, 49 μL of the polymerase chain reaction (PCR) mix was pipetted into the 0.2 mL microcentrifuge tube. 1 μL of the cell solution was then added to the PCR mix. In the PCR procedure, initial denaturation, second denaturation, annealing, initial elongation and final elongation were conducted at a temperature of 94 °C for 1 min (1 cycle), 94 °C for 1 min (30 cycles), 55 °C for 1 min (30 cycles), 72 °C for 5 min (30 cycles) and 72 °C for 10 min (1 cycle), respectively. PCR was employed to amplify a specific region of the 16S rRNA gene of unknown bacteria. The reaction set-up was used to generate the DNA sequence of the amplified DNA. The actual DNA sequencing gel was run and read at the 1st BASE Laboratories, Taman Serdang Perdana, Malaysia. Then, the basic local alignment search tool (BLAST) analysis of 16S rRNA gene sequence was employed to search the top match sequences in the database. 

### 3.6. Antibacterial Appraisal

Agar nutrient was first prepared by mixing 14 g agar powder nutrient with 500 mL of ultrapure water. In the present work, the antibacterial capability of the synthesized AgNPs was examined using the paper disk assay method [47]. About $5\times 10^{15}$ colonies of bacterial cultures were taken and about 0.1 mL of inoculum was spread on each agar plate. In the preparation, AgNPs solution was prepared by mixing the AgNO_3_ solution with a concentration of 0.15 M and plant extracts (1:1) in a 50 mL plastic tube. In this test, the concentration of AgNPs was around 200 mg·L^−1^ prepared by the dilution procedure. For comparison purpose, AgNO_3_ solution, ultrapure water and plant extracts were prepared. Filter paper (2 mm in diameter) was steeped in AgNPs solution for 1 min and then placed on the agar plate. In addition, other filter papers were steeped in AgNO_3_ solution, ultrapure water and plant extracts and gently placed on the plates. For monitoring the zones of inhibition of AgNPs, AgNO_3_, ultrapure water and plant extracts, all plates were subsequently incubated at 37 °C for 24 h. This test was performed in three replicates to ensure its result repeatability. 

### 3.7. Characterization

The concentration of AgNPs was examined using the ICPOES (710 ICPOES No. my13270004, purchased from Agilent, Santa Clara, CA, USA) operated at a power of 1200 W with Argon flow rate of 15 L·min^−1^. UV-vis spectra were monitored using the UV-Vis spectrometer (101N4110104, purchased from PerkinElmer, Waltham, MA, USA) operated at a resolution of 1 nm, a scan speed of 960 nm·min^−1^ and an electromagnetic wavelength in the range of 300 to 700 nm. FTIR spectra were investigated using the FTIR spectrometer (Frontier-GPOB model 96046, purchased from PerkinElmer, Waltham, MA, USA) at a spectrum wavelength in the range of 400 to 4000 cm^−1^ at a resolution of 4 cm^−1^ and accumulations of 10 scans at room temperature. In the preparation, a pellet form was prepared by mixing AgNPs with potassium bromide (1:100). FESEM and EDX were examined by means of the field emission scanning electron microscopy (FESEM Supra 35VP, purchased from ZEISS, Oberkochen, Baden-Württemberg, Germany) and SEM-EDX (S-3400N, purchased from HITACHI, Chiyoda, Tokyo, Japan), respectively. The nanoparticles size was obtained by randomly measuring the selected samples from the FESEM data following the procedure established elsewhere [7,48]. 

## 4. Conclusions

This work characterized and evaluated the use of extracts of *Cyperus rotundus*, *Eleusin indica*, *Euphorbia hirta*, *Melastoma malabathricum*, *Clidemia hirta* and *Pachyrhizus erosus* to synthesize AgNPs against bacteria available in the river and sewage treatment plant. Spherical AgNPs can be synthesized when *Cyperus rotundus*, *Eleusin indica*, *Pachyrhizus erosus* and *Clidemia hirta* were employed. On the contrary, AgNPs synthesized using *Euphorbia hirta* and *Melastoma malabathricum* extracts were non-uniform in shape. In addition, about 72% to 91% efficiencies of AgNO_3_ were successfully extracted to form AgNPs with the highest synthesis efficiency obtained when *Eleusin indica* extract was used. In general, this study has also confirmed that all synthesized AgNPs have antibacterial capability against all investigated bacteria. Moreover, it is interesting to report that *Melastoma malabathricum* and *Clidemia hirta* extracts have antibacterial capability against all bacteria isolated from STP and rivers. Further studies need to be carried out in order to validate the effects of physio-chemical parameters to synthesize AgNPs using the presently studied plant extracts. In addition, the cytotoxicity investigations are also crucial for further antibacterial characterization in the future work. 

## Figures and Tables

**Table 1:** Biochemical contents of all employed plants.

Leaf Extract	Biochemical Content	References
*Cyperus rotundus*	Essential oils, alkaloids, flavonoids, terpenoids, chromones, phenylpropanoids, phenolic acids and iridoides	[15]
*Eleusin indica*	Flavonoids, terpenoids, phenols, phenolic glycosides, saponins, cyanogenic glycosides, unsaturated lactones, glucosinolates and C-glycosylflavones	[19]
*Euphorbia hirta*	Alkaloids, saponins, terpenoids, flavonoids, tannins phenolic acids and amino acids	[21]
*Melastoma malabathricum*	Naringenin, terpenoids, kaempferol, flavonoids, kaempferol-3-*O*-d-glucoside and ethyl acetate	[22,25]
*Clidemia hirta*	Flavonoids, terpenoids, phenolics and saponins	[23]
*Pachyrhizus erosus*	Rotenone, cysteine protease, albumins, globulins, prolamins and glutelins.	[18,20,24]

**Table 2:** Possible compounds of AgNPs synthesized using different plant extracts.

Plants Name	Plant Extracts (cm^−1^)	AgNPs (cm^−1^)	Possible Compound
*Cyperus rotundus*	3363	-	–OH
	2976	2976	–CH
	1715	1715	C=O
	1248	1262	C–O–C
	1099	1099	P–O
	726	726	N–H
*Eleusin indica*	3339	-	–OH
	2919	-	–CH
	1709	1709	C=O
	1250	1250	C–O–C
	1021	1021	P–O
	726	721	N–H
*Euphorbia hirta*	3312	3340	–OH
	2921	2921	–CH
	1715	1715	C=O
	1100	1100	P–O
	726	-	N–H
*Melastoma malabathricum*	3321	-	–OH
	2925	2941	–CH
	1714	1714	C=O
	1246	1250	C–O–C
	1018	1020	P–O
	724	724	N–H
*Clidemia hirta*	2973	2973	–CH
	1716	1716	C=O
	1266	1266	C–O–C
	1018	1018	P–O
	726	726	N–H
*Pachyrhizus erosus*	2969	2969	–CH
	1714	1714	C=O
	1266	1266	C–O–C
	1019	1019	P–O
	725	725	N–H

**Table 3:** Shape and size of AgNPs synthesized using all plants.

Plant Extracts	Shape	Size (nm)
*Cyperus rotundus*	Spherical	20.5 ± 9.6
*Eleusin indica*	Spherical	55.0 ± 24.1
*Euphorbia hirta*	Irregular	56.25 ± 21.8
*Melastoma malabathricum*	Irregular	108.35 ± 36.3
*Clidemia hirta*	Irregular	57.4 ± 24.2
*Pachyrhizus erosus*	Spherical	40.6 ± 10.8

**Table 4:** Zone of inhibition of AgNPs, AgNO_3_, water and plant extracts.

Plant	Bacteria	AgNPs (mm)	AgNO_3_ (mm)	Water (mm)	Plant Extracts (mm)
*Cyperus rotundus*	*E. coli*	15.00 ± 0.36	1.07 ± 0.06	-	-
	*B. cereus*	10.00 ± 0.26	0.93 ± 0.12	-	-
	*C. haemolyticum* UDIN2	8.00 ± 0.26	1.00 ± 0.00	-	-
	*C. haemolyticum* UDIN3	10.30 ± 0.23	1.19 ± 0.25	-	-
	*C. haemolyticum* UDIN1	9.00 ± 0.25	1.00 ± 0.17	-	-
*Eleusin indica*	*E. coli*	12.30 ± 0.06		-	-
	*B. cereus*	9.70 ± 0.30		-	-
	*C. haemolyticum* UDIN2	8.70 ± 0.06		-	-
	*C. haemolyticum* UDIN3	9.30 ± 0.15		-	-
	*C. haemolyticum* UDIN1	12.00 ± 0.17		-	-
*Euphorbia hirta*	*E. coli*	12.30 ± 0.50		-	-
	*B. cereus*	9.00 ± 0.17		-	-
	*C. haemolyticum* UDIN2	9.00 ± 0.11		-	-
	*C. haemolyticum* UDIN3	13.30 ± 0.30		-	-
	*C. haemolyticum* UDIN1	12.00 ± 0.17		-	-
*Melastoma malabathricum*	*E. coli*	1.60 ± 0.10		-	-
	*B. cereus*	1.40 ± 0.17		-	-
	*C. haemolyticum* UDIN2	1.23 ± 0.31		-	0.27 ± 0.46
	*C. haemolyticum* UDIN3	1.20 ± 0.17		-	0.40 ± 0.32
	*C. haemolyticum* UDIN1	1.17 ± 0.50		-	0.53 ± 0.47
*Clidemia hirta*	*E. coli*	1.60 ± 0.10		-	-
	*B. cereus*	1.03 ± 0.06		-	-
	*C. haemolyticum* UDIN2	1.06 ± 0.06		-	0.87 ± 0.1
	*C. haemolyticum* UDIN3	1.27 ± 0.29		-	0.9 ± 0.17
	*C. haemolyticum* UDIN1	1.33 ± 0.38		-	0.8 ± 0.00
*Pachyrhizus erosus*	*E. coli*	11.00 ± 0.05		-	-
	*B. cereus*	11.00 ± 0.10		-	-
	*C. haemolyticum* UDIN2	15.00 ± 0.10		-	-
	*C. haemolyticum* UDIN3	9.00 ± 0.10		-	-
	*C. haemolyticum* UDIN1	15.00 ± 0.10		-	-

**Table 5:** Antibacterial capability of AgNPs against several bacteria.

Species	Bacteria Name	Shape	Size (nm)	Inhibition Zone (mm)	References
Gram-negative	*Proteus vulgaris*	Cubic	19	8 ± 0.0	[9]
	*Serratia marcescens*			8 ± 0.8	
	*Salmonella typhi*			8 ± 0.3	
	*Vibrio parahaemolyticus*	Spherical	97	25 ± 1.0	[10]
	*Salmonella enterica*			12 ± 0.6	
	*Vibrio anguillarum*	Spherical and irregular	7–31 and 7–19	11.2 ± 0.5	[12]
	*Vibrio alginolyticus*			10.8 ± 1.4	
	*Aeromonas punctata*			10.5 ± 0.7	
	*Vibrio parahaemolyticus*			11.0 ± 1.3	
	*Escherichia coli*	Spherical	9 ± 2	9 ± 1.0	[13]
	*Pseudomonas aeruginosa*			9 ± 0.5	
Gram-positive	*Enterococcus faecalis*	Spherical	10–60	2.5–6.2	[8]
	*Staphylococcus epidermidis*	Cubic	19	7.5 ± 0.0	[9]
	*Methicillin-resistant*			6	
	*Bacillus subtilis*			6	
	*Streptococcus faecalis*			6	
	*Bacillus anthracis*	Spherical	97	15 ± 0.8	[10]
	*Staphylococcus aureus*	Spherical	20–30	7–21	[11]
	*Staphylococcus aureus*	Spherical	9 ± 2	11 ± 0.6	[13]
